# Adaptive Motion Artifact Reduction Based on Empirical Wavelet Transform and Wavelet Thresholding for the Non-Contact ECG Monitoring Systems

**DOI:** 10.3390/s19132916

**Published:** 2019-07-01

**Authors:** Xiaowen Xu, Ying Liang, Pei He, Junliang Yang

**Affiliations:** 1 School of Physics and Electronics, Central South University, Changsha 410083, China; 2 School of Basic Medical Science, Central South University, Changsha 410013, China

**Keywords:** electrocardiogram, empirical wavelet transform, wavelet thresholding, motion artifact removal, non-contact ECG monitoring system

## Abstract

Electrocardiogram (ECG) signals are crucial for determining the health status of the human heart. A clean ECG signal is critical in analysis and diagnosis of heart diseases. However, ECG signals are often contaminated by motion artifact noise in the non-contact ECG monitoring systems. In this paper, an ECG motion artifact removal approach based on empirical wavelet transform (EWT) and wavelet thresholding (WT) is proposed. This method consists of five steps, namely, spectrum preprocessing, spectrum segmentation, EWT decomposition, wavelet threshold denoising, and EWT reconstruction. The proposed approach was used to process real ECG signals collected by the non-contact ECG monitoring equipment. The results of quantitative study and analysis indicate that this approach produces a better performance in terms of restorage of QRS complexes of the original ECG with reduced distortion, retaining useful information in ECG signals, and improvement of the signal to noise ratio (*SNR*) value of the signal. The output results of the practical ECG signal test show that motion artifact in the real recorded ECG is effectively filtered out. The proposed method is feasible for reducing motion artifacts from ECG signals, whether from simulation ECG signals or practical non-contact ECG monitoring systems.

## 1. Introduction

The electrocardiogram (ECG) signal is a vital tool to reflect the electrical activity of heart muscles. ECG signals are one of the most important biological signals, which can be used clinically to observe the activity of the human heart, screen for cardiovascular diseases, and evaluate cardiac and cardiovascular functions [1,2]. Recently, non-contact ECG monitoring systems have begun to be widely used to monitor biomedical signals. However, during ECG signal collection in ECG monitoring systems, ECG signals are often corrupted with motion artifact noise due to the unstable contact between the skin and the surface of the electrodes, muscle contraction and breathing. This unexpected noise in ECG signals can produce detrimental effects, which are not conducive to the diagnosis of the heart condition [3,4]. Thus, motion artifact elimination from ECG signals is essential for accurate monitoring and diagnosis of heart health.

Several approaches to motion artifact removal in ECG signals have been previously reported, including adaptive filter (AF) [4,5,6], wavelet transform (WT) [7,8] and empirical mode decomposition (EMD) [9,10]. Generally, the practical method of removing motion artifacts is to employ a low-pass filter to process the signal. The adaptive filtering can eliminate the noise without signal distortion by using the deterministic function as a reference signal [11,12,13,14]. However, the adaptive filtering to remove ECG motion artifacts is limited due to the need for accurate reference signals as a prerequisite.

Wavelet transform is one of the most widely used denoising methods in signal processing in various fields [15,16]. Daqrouq [7] proposed the zero-phase high pass FIR equiripple filtering approach to reduce the baseline wander in ECG signals. Hashim et al. [17] used the wavelet threshold method to reduce motion artifact noise in ECG signals. He et al. [18] employed the sliding window to analyze signal complexity. Nagai et al. [19] used the stationary wavelet transform (SWT) to remove motion artifact superimposed on ECG signals when using non-contact capacitively coupling electrodes. However, the wavelet transform method requires the selection of appropriate wavelet functions and thresholds, which makes the wavelet method non-adaptive.

To solve the shortcomings of wavelet transform without adaptability, Huang et al. [20] proposed an empirical mode decomposition method, which is an adaptive method for decomposing signals into a finite number of intrinsic mode functions (IMFs). Lee et al. [9] used EMD to detect motion artifacts of Holter ECG signals. Blanco-Velasco et al. [10] proposed an ECG enhancement method based on the EMD to remove high-frequency noise and baseline wander. However, mode mixing is a common problem in the EMD method, which causes IMFs to be extracted incorrectly [21].

An adaptive wavelet analysis method based on the empirical wavelet transform (EWT) has been proposed for feature vectors extracted in two wavelet matrices [22]. The EWT method has been widely used in bearing fault diagnosis [23,24], wind speed prediction [25,26], disease classification detection [27], transmission line short-circuit fault detection [28], and ECG denoising [29]. The EWT method showed a better performance than the EMD method when using a filter to reduce power-line interference and correct baseline drift [29]. In this paper, we propose a method based on empirical wavelet transform and wavelet thresholding (EWT-WT) to remove the motion artifact from the practical ECG signals. Simulated signals and real recorded ECG signals are used to verify the feasibility of the algorithm. Compared with the discrete wavelet transform (DWT) and the EMD motion artifact removal approaches, the EWT- WT method shows better performance in terms of restorage of the original ECG QRS complexes and improvement of the signal to noise ratio (*SNR*) [30,31,32] value of the ECG signals.

## 2. Proposed Method

### 2.1. Databases

#### 2.1.1. Simulated ECG Data

The standard MIT-BIH arrhythmia database contains a total of 48 ECG records of a duration of 30 min each [33]. The ECG records 100, 103, 104, 109, 123, 201, 208, 209, 213 and 219 and the dataset ‘em’ from the MIT-BIH Noise Stress Test Database [34] were used for simulation purpose. These signals include time-varying QRS morphology, both normal and abnormal ECG beats. The noisy ECG records were obtained by adding motion artifacts to the ECG signals at different levels of *SNR*.

#### 2.1.2. Real ECG Data

**Figure 1:**
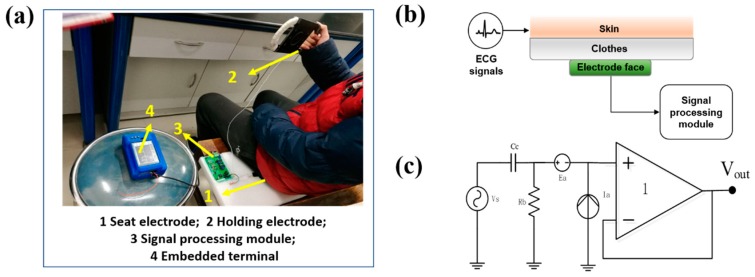
(**a**) Non-contact ECG monitoring system; (**b**) Schematic diagram of the capacitive coupling electrode; (**c**) Equivalent circuit diagram of the electrode.

We evaluated the feasibility of the proposed method to reduce the motion artifact from real ECG signals acquired by a non-contact ECG monitoring system as shown in Figure 1a. All data were collected anonymously. Each participant was given verbal consent before the experiment. The volunteer sat on the seat electrode and held the holding electrode composed of conductive fabric to collect the standard II lead ECG signal. The electrodes of this system are designed based on the principle of capacitive coupling [35,36,37,38,39], as shown in Figure 1b. The human skin and the conductive fabric on the surface of the electrode form the two plates of the coupling capacitor, and the insulating medium of the garment coupling capacitor is an insulator layer such as clothing. The equivalent circuit diagram of the capacitor electrode is shown in Figure 1c. The surface charge of the capacitive electrode will vary with the change of skin surface potential. The ECG signal can be collected by capacitive coupled electrodes through clothing. The ECG signals were recorded from three volunteers in both static and dynamic states. During the recording, the volunteers held the fabric electrode in the right hand and sat on the seat electrode. The static state required the volunteers keep their limbs and hands still, while the volunteers were free to turn their hands at the dynamic states. 

### 2.2. Theory of Empirical Wavelet Transform

The empirical wavelet transform (EWT) methodology is a fully adaptive, data-driven and signal processing method, which combines the mathematical theory of the wavelet transform method and the adaptability of the empirical mode decomposition method [22]. The detailed calculation process of EWT was introduced in [22]. Here, we briefly describe the specific process of EWT as follows:

*1)*  Compute the Fast Fourier Transform (FFT) of the signal x(t) to obtain the spectrum X(ω). Detect the local maxima $\omega_{n}$ in the spectrum and select the top M values in descending order as MA.
*2)*  Perform the Fourier spectrum segmentation. We assume that the Fourier support [0,π] is segmented into N (N<M) contiguous segments, and maintain the first *N*−1 local maxima $MI=\left\{\omega_{1},\omega_{2},\omega_{3},\ldots ,\omega_{N-1}\right\}$ (excluding 0 and π). Centered around each $\omega_{n}$, we define a transition phase $T_{n}$ of width $2\tau_{n}$. The boundary of each segment $\left(\Lambda_{i}\right)$ is defined as the center of two consecutive local maxima values:
  (1) $$\Lambda_{i}=\frac{\omega_{i}+\omega_{i+1}}{2}$$
  The spectrum boundary is: $\Lambda =\{\Lambda_{1},\Lambda_{2},\Lambda_{3},\ldots ,\Lambda_{N-1}\}$;
*3)*  Based on the detected spectral boundaries, we choose the Meyer wavelet as the basis function. The adaptive wavelet filter bank which consists of a low-pass filter (scaling function) and a band-pass filter (wavelet function) is designed by using Equations (2) and (3), respectively.
  (2) $$\Phi \left(\omega \right)=\{\begin{array}{c} 1,\;\;\left|\omega \right|\leq \omega_{n}-\tau_{n}\\ \cos\left[\frac{\pi }{2}\beta \left(\frac{1}{2\tau_{n}}\left(\left|\omega \right|-\omega_{n}+\tau_{n}\right)\right)\right],\;\omega_{n}-\tau_{n}\leq \left|\omega \right|\leq \omega_{n}+\tau_{n}\\ 0,\;\;\mathrm{otherwise} \end{array}$$
  (3) $$\Psi_{n}\left(\omega \right)=\{\begin{array}{c} 1,\;\;\;\;\omega_{n}+\tau_{n}\leq \left|\omega \right|\leq \omega_{n+1}-\tau_{n+1}\\ \cos\left[\frac{\pi }{2}\beta \left(\frac{1}{2\tau_{n+1}}\left(\left|\omega \right|-\omega_{n+1}+\tau_{n+1}\right)\right)\right],\;\omega_{n+1}-\tau_{n+1}\leq \left|\omega \right|\leq \omega_{n+1}+\tau_{n+1}\\ \sin\left[\frac{\pi }{2}\beta \left(\frac{1}{2\tau_{n}}\left(\left|\omega \right|-\omega_{n}+\tau_{n}\right)\right)\right],\;\omega_{n}-\tau_{n}\leq \left|\omega \right|\leq \omega_{n}+\tau_{n}\\ 0,\;\;\mathrm{otherwise} \end{array}$$
  Correctly select the parameter $\tau_{n}$ to ensure that EWT is a tight frame. β(x) is defined as follows [22]:
  (4) $$\beta \left(x\right)=x^{4}\left(35-84x+70x^{2}-20x^{3}\right)$$
  Define EWT after exporting the scaling function and the empirical wavelet. The approximation coefficients, $W_{f}^{\varepsilon }\left(0,t\right)$, is the inner product of the signal and the scaling function:
  (5) $$W_{f}^{\varepsilon }\left(0,t\right)=f,\phi_{1}=\int f\left(\tau \right)\bar{\phi_{1}\left(\tau -t\right)}d\tau$$
  The detail coefficients, which are given by the inner product of the signal and the empirical wavelet, are presented below:
  (6) $$W_{f}^{\varepsilon }\left(n,t\right)=\langle f,\psi_{n}\rangle =\int f\left(\tau \right)\bar{\psi_{n}\left(\tau -t\right)}d\tau$$
  The empirical mode of signal decomposition is as follows:
  (7) $$f_{0}\left(t\right)=W_{f}^{\varepsilon }\left(0,t\right)*\phi_{1}\left(t\right)$$
  (8) $$f_{k}\left(t\right)=W_{f}^{\varepsilon }\left(k,t\right)*\psi_{k}\left(t\right)$$
*4)*  The extracted pattern is defined as the output of the scaling function and the wavelet function.

### 2.3. The Proposed Method

**Figure 2:**
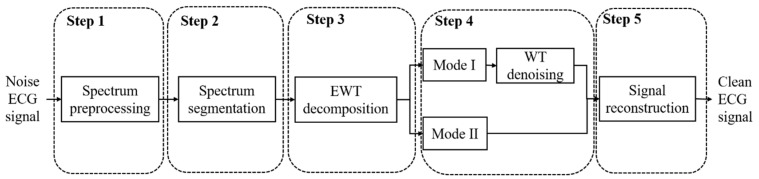
The flowchart of the EWT-WT motion artifact removal method.

In this section, we will introduce the proposed method based on EWT-WT. The flowchart of the proposed method is shown in Figure 2. It consists of five parts, namely, spectrum preprocessing, spectrum segmentation, EWT decomposition, WT denoising, and EWT reconstruction. The detailed discussion is introduced in the following description. 

Step 1: Spectrum preprocessing. The noisy ECG signal x(*t*) was generated by adding motion artifact noise m(*t*) to a clean ECG c(*t*).

(9) x(t)=m(t)+ c(t)

In order to obtain the appropriate spectral-boundaries, the FFT of the signal x(*t*) was performed to obtain the spectrum X(*ω*).

(10) X(ω)=FFT[x(t)]

**Figure 3:**
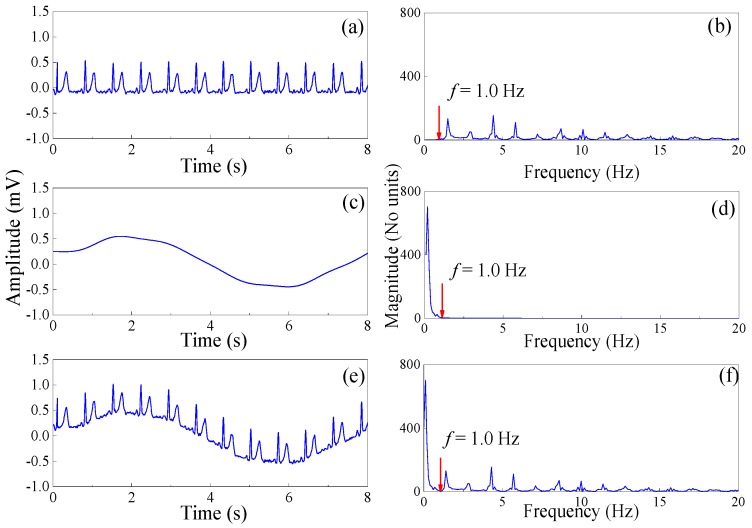
(**a**) The time domain diagram of the clean ECG. (**b**) The frequency domain diagram of the clean ECG. (**c**) The time domain diagram of the motion artifact noise. (**d**) The frequency domain diagram of the motion artifact noise. (**e**) The time domain diagram of the noisy ECG signal. (**f**) The frequency domain diagram of the noisy ECG signal.

The time and frequency domain diagram of the clean ECG, the motion artifact noise, and the noisy ECG signals are shown in Figure 3. It can be seen that the spectral boundary between clean ECG and the motion artifact noise is *f* = 1.0 Hz (Figure 3).

**Figure 4:**
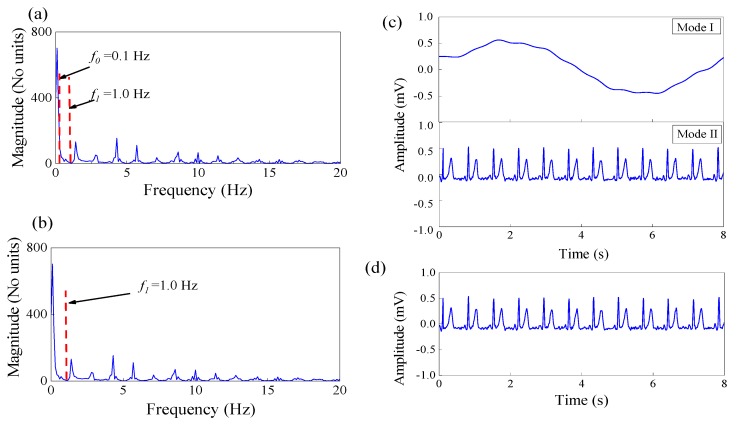
Segmentation of the Fourier spectrum of the ECG signal contaminated by motion artifacts. (**a**) Original spectral-boundary and (**b**) final spectral-boundary; (**c**) Modes extracted by the EWT and (**d**) EWT reconstructs ECG signals.

Step 2: Spectrum segmentation. We use the local maxmin method to specify the boundary as the middle between successive maxima. We set the Fourier support to be segmented into *N* (*N* = 3) contiguous segments, and maintain the first *2(N−1)* local maxima *ΜΙ* = {*ω*_1_, *ω*_2_}. Figure 3 shows that the spectral boundary of clean ECG and the motion artifact noise is *f* = 1.0 Hz. Figure 4 shows spectrum segmentation of the ECG signal contaminated by motion artifacts. The first boundary (*f*_0_ = 0.1 Hz) has no practical meaning, as shown in Figure 4a. We set the spectral boundary *f* = 1.0 Hz as the second boundary. Thus, the final spectral boundary *f*_1_ = 1.0 Hz divides the signal spectrum into two modes, noisy dominant $x_{N}\left(t\right)$ and useful ECG $x_{c}\left(t\right)$ signal, as shown in Figure 4b. 

Step 3: EWT decomposition. In the proposed method, the EWT algorithm is used to eliminate the motion artifact noise from ECG signals, and decompose the noise-containing ECG signal (ECG + motion artifact) into two modes, and the empirical mode of signal decomposition is as follows:

(11) $$f_{k}\left(t\right)=W_{f}^{\varepsilon }\left(k,t\right)*\psi_{k}\left(t\right)$$

Here, the $W_{f}^{\varepsilon }\left(k,t\right)$ represents the corresponding approximation coefficients and the detail coefficients. The *ψ_k_(t)* represents the corresponding band-pass filter (wavelet function). The detailed calculation process of EWT has been introduced in [22].

The signal is processed by a bandpass filter bank and then decomposed to two empirical modes. The decomposition results are shown in Figure 4c.

Step 4: WT denoising. For the two empirical mode components, Mode I $x_{N}\left(t\right)$ is dominated by motion artifacts and may contain partial components of the ECG signal, while Mode II $x_{c}\left(t\right)$ contains the main useful ECG signal. 

For Mode I, the motion artifact noise is eliminated by using the ‘heursure’ wavelet threshold method with an optimized threshold [7,13,14,15,16].

We assume that the number of input signals is N, and:

$$\alpha =\frac{\sum_{i}^{N}{\left|x_{i}\left(n\right)\right|}^{2}-N_{i}}{N_{i}},\;\beta =\sqrt{\frac{1}{N_{i}}\left({\frac{\ln N_{i}}{\ln2}}^{3}\right)}$$

Then,

(12) $$Thr=\{\begin{array}{c} \sigma \sqrt{2\log N},\;\;\alpha <\beta \\ \sqrt{y_{i}\left(\theta_{min}\right)},\;\;\alpha \geq \beta \end{array}$$

where *θ_min_* is the *θ* value at which the risk reaches a minimum. Thus, the WT denoising results are obtained by the Equation (13).

(13) $$\bar{x_{N}}\left(t\right)=Thr\left[x_{N}\left(t\right)\right]$$

Step 5: Signal reconstruction. After wavelet threshold denoising, Mode I is added to Mode II.

(14) $$\bar{x}\left(t\right)=\bar{x_{N}}\left(t\right)+\;x_{c}\left(t\right)$$

The combined signal is reconstructed by the inverse empirical wavelet transform.

(15) $$y\left(t\right)=i\mathrm{EWT}\left[\bar{x}\left(t\right)\right]$$

The detailed calculation process of the inverse empirical wavelet transform has been introduced in [22]. Finally, the output ECG signal after motion artifact elimination is obtained, as shown in Figure 4d.

## 3. Results

The performance of the EWT-WT method was evaluated using ECG signals from the MIT-BIH database. We selected 10 sets of ECG signals in the MIT-BIH arrhythmia database as the noise-free ECG signal, and the ‘em’ signal in the MIT-BIH Noise Stress Test Database as the motion artifact noise. A synthetic ECG signal with motion artifact noise was obtained by adding the motion artifact to the noise-free ECG signal. The performance of the proposed EWT-WT based method to remove motion artifact was compared with the DWT [8], EMD [10] and EWT [29] methods.

**Figure 5:**
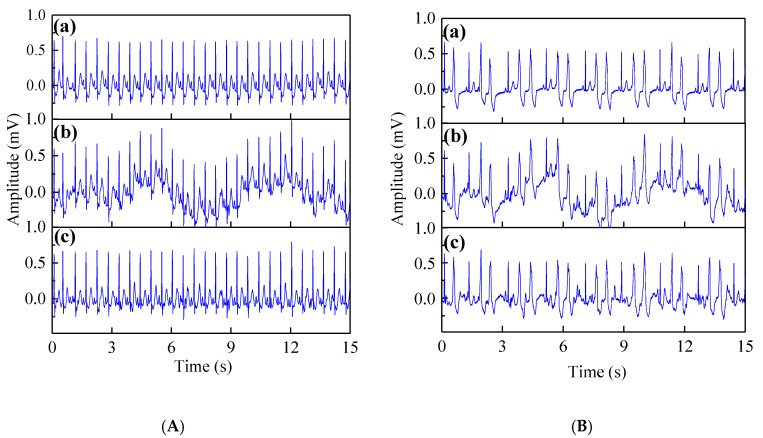
(**A**) The denoising results of the EWT-WT motion artifact removal method on normal beat record 213. (**B**) The denoising results of the EWT-WT motion artifact removal method on abnormal beat record 208. (**a**) Clean ECG signal; (**b**) Noisy ECG signal; (**c**) Denoised ECG.

Firstly, the qualitative performance analysis of the proposed method was carried out. Figure 5A presents the results for the normal beat record 213 and Figure 5B indicates the results for the abnormal beat record 208. The results show the proposed method can effectively remove motion artifacts for the normal and arrhythmia ECG signals.

**Figure 6:**
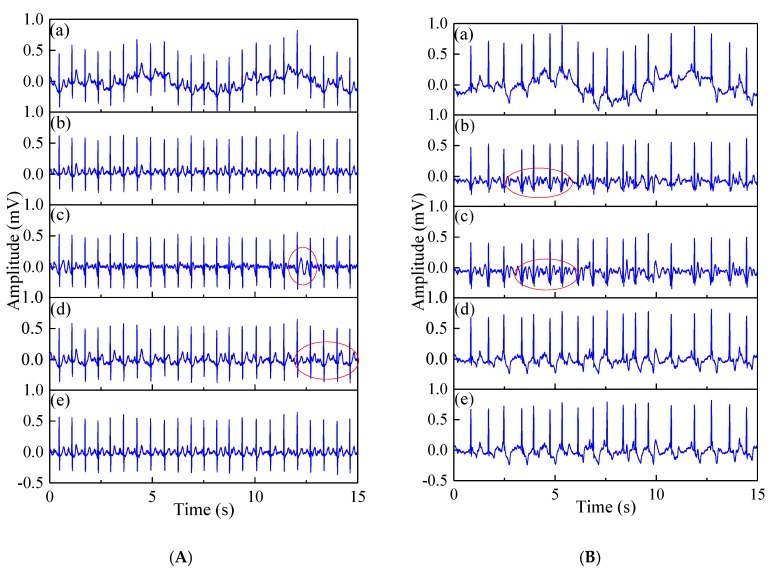
The comparison of the denoising results of different motion artifact removal methods on normal beat record 209 (**A**) and arrhythmia record 219 (**B**); (**a**) Noisy ECG signal; (**b**) DWT; (**c**) EMD; (**d**) EWT; (**e**) EWT-WT. The red circle indicates partial distortion of the signal.

Figure 6 presents the qualitative analysis of the normal beat signal (record 209) and the arrhythmia signal (record 219) through various noise removal methods, respectively. Both normal beat record 209 and arrhythmia record 219 were added to motion artifacts at *SNR* = 0 (Figure 6).

Then, the efficacy of the proposed method was evaluated using four performance metrics namely correlation coefficient (*Corr*), mean square error (*MSE*), percentage root mean square difference (*PRD*) and output *SNR* improvement (*SNR_imp_*). The performance parameters considered for evaluation can be expressed as follows.

The correlation coefficient (*Corr*):

(16) $$Corr=\frac{Cov\left(x\left(i\right),y\left(i\right)\right)}{\sqrt{D\left(x\left(i\right)\right)}\sqrt{D\left(y\left(i\right)\right)}}\;$$

The mean square error (*MSE*):

(17) $$MSE=\frac{1}{N}\sum_{i=1}^{i=N}{\left(x\left(i\right)-y\left(i\right)\right)}^{2}$$

The percentage root mean square difference (*PRD*):

(18) $$PRD=100\times \sqrt{\frac{\sum_{i=1}^{i=N}{\left(x\left(i\right)-y\left(i\right)\right)}^{2}}{\sum_{i=1}^{i=N}{\left(x\left(i\right)\right)}^{2}}}\;$$

The output *SNR* improvement (*SNR_imp_*):

(19) $$SNR_{imp}=10\times \log_{10}\left(\frac{\sum_{i=1}^{i=N}{\left(\bar{x}\left(i\right)-x\left(i\right)\right)}^{2}}{\sum_{i=1}^{i=N}{\left(y\left(i\right)-x\left(i\right)\right)}^{2}}\right)$$

Here, x(*i*) is the original ECG signal, $\bar{x}\left(i\right)$ is the infected ECG signal by the motion artifact noises, y(*i*) is the denoised ECG signal, and *N* is the length of the ECG signal.

**Figure 7:**
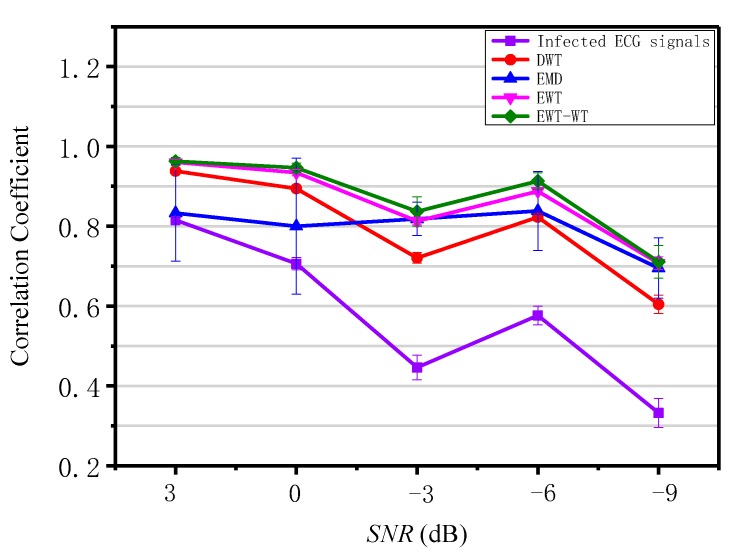
Comparison of correlation coefficient for EWT-WT with DWT, EMD and EWT.

In order to verify the performance of the EWT-WT motion artifact removal methodology, we select 10 sets of ECG signals from the MIT-BIH Arrhythmia Database as original ECG signals, and added motion artifacts with different signal-to-noise ratios to simulate infected ECG signals. The output of denoised ECG signals and the input of infected ECG signals were compared to the corresponding original ECG signal, while the correlation coefficient was used to verify the performance of EWT-WT motion artifact removal methodology. The test results are shown in Figure 7.

Table 1, Table 2 and Table 3 show the results of filtering of the motion artifacts at *SNR* = 0 for ECG signals from the MIT-BIH database. Compared with DWT-based, EMD-based and EWT-based motion artifact removal method, the proposed method provides an efficient solution to deal with the motion artifact noise.

**Figure 8:**
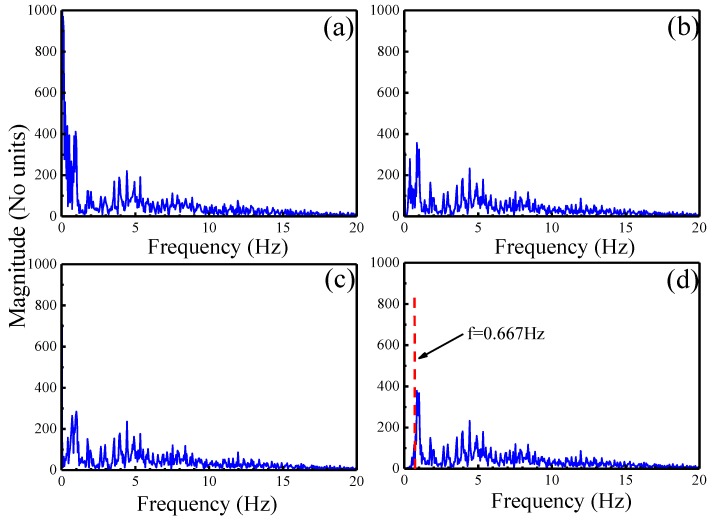
The frequency domain diagram of the denoised signals for real ECG signals using various motion artifact removal methods. (**a**) Original ECG signal; (**b**) DWT; (**c**) EMD; (**d**) EWT-WT.

**Figure 9:**
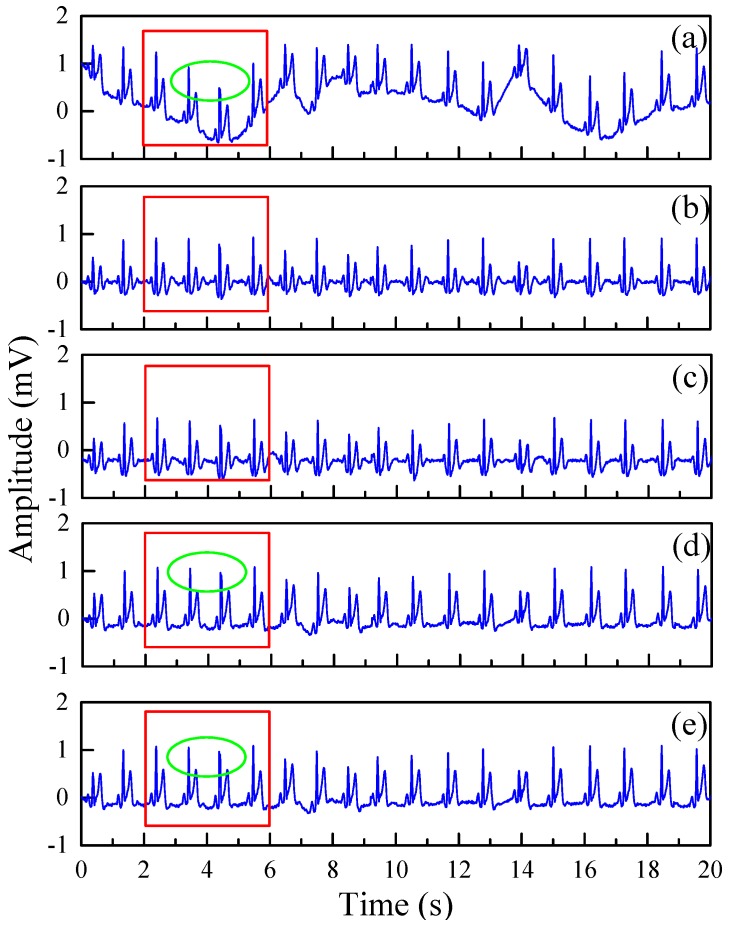
The results of different motion artifact removal methods for real ECG signals. (**a**) Original ECG signals; (**b**) DWT; (**c**) EMD; (**d**) EWT; (**e**)EWT-WT.

**Figure 10:**
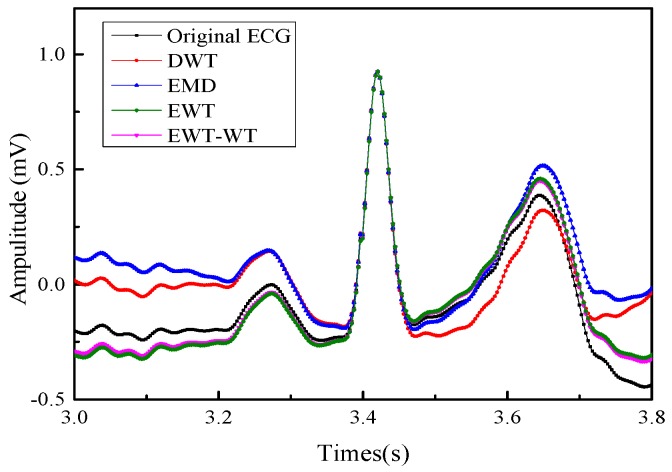
The original ECG and the denoised ECG using various methods for a real ECG.

Figure 8 shows the frequency domain diagram of the denoising results using DWT, EMD, and EWT-WT methods for real ECG signals. Figure 9 shows the results of different motion artifact removal methods for real ECG signals. The red line area indicates that the proposed EWT-WT based method can efficiently remove the effects of motion artifact and maintain all the characteristic details of the ECG signals. The ECG signals denoised by the DWT and EMD based approaches show partial distortion. Comparison results of the WT and EWT-WT methods for real ECG signals are shown in a green colored circle in Figure 9d,e. In order to observe the retained useful information more intuitively, we compared the ECG details of the original signal and the denoised signal. Figure 10 presents the original ECG signals and the denoised ECG signals using various methods for a real ECG signal.

## 4. Discussion

### 4.1. Qualitative Analysis

As we can see from Figure 5, whether for normal beat ECG signals or arrhythmia ECG signals, the EWT-WT method can effectively remove the noise and preserve the meaningful details such as the P-wave, T-wave and QRS complex.

Figure 6 shows the denoising results using different motion artifact removal methods on normal beat record 209 and abnormal beat record 219. The results of the DWT, EMD and WT methods show obvious distortion of the ECG signals, as shown in the red circle. However, the proposed EWT-WT motion artifact removal approach can efficiently remove the motion artifact from the ECG signals, and maintain the characteristic details of the ECG signals. 

### 4.2. Quantitative Analysis

The infected ECG signals were processed by DWT, EMD, EWT and EWT-WT methods. The correlation coefficient is commonly used as a performance parameter in the study of the motion artifact removal for ECG signals [40]. The correlation coefficient between the denoised ECG signal and the original ECG signal was obtained, as shown in Figure 7. It can be seen that the average correlation coefficient of the EWT-WT method is higher than that of the DWT, EMD and EWT methods, which indicates that the EWT-WT method has better restorage for the original ECG signal. 

Table 1, Table 2 and Table 3 illustrate the *MSE*, the *PRD* and the *SNR_imp_* of the denoised ECG signal by DWT, EMD, EWT and EWT-WT methods. The *MSE* is one of the significant metrics to evaluate the performance of an algorithm. The small *MSE* level indicates better recovery for the original signal. The MSE comparison of various denoising methods is summarized in Table 1. The MSE of denoised signals by the EWT-WT method is less than that of DWT, EMD and EWT methods. It indicates that the EWT-WT motion artifact removal method has more effective recovery ability for the original ECG signal. The *PRD* determines the degree of distortion, which occurs in the denoised signal. It defines the noise reduction ability of the filtering method without losing any critical information. The small *PRD* value indicates lower distortion and better recovery of the original signal. The *PRD* of the denoised ECG signals using the EWT-WT, DWT, EMD and EWT based motion artifact removal methods is summarized in Table 2. The results show that the EWT-WT motion artifact removal method has the smallest *PRD* value for all recorded signals, which suggests that the EWT-WT method has better performance in restorage of the original ECG signal with less distortion. The *SNR_imp_* denotes the improvement in *SNR* value of a signal by the filtering process and can be used to quantify the efficacy of the filter for motion artifact elimination. The *SNR_imp_* comparison of the denoising results of the motion artifacts in ECG signals is presented in Table 3. It illustrates that the EWT-WT based motion artifact removal method has better *SNR_imp_* than the DWT, EMD and EWT methods for ECG signals, which indicates better performance of the proposed method in improving the *SNR* value of the signal and effectively removing the motion artifact noise. The proposed method outperforms the others in terms of *MSE*, *PRD* and *SNR_imp_*.

### 4.3. Real ECG Signals Testing

Figure 8 shows the spectrum corresponding to the real ECG signals after denoising using different methods. The spectrum of motion artifacts in the original ECG signal mainly concentrates on the first peak. The EWT-WT method can effectively remove the information of the first peak, but the denoising results using DWT and EMD still contain part of the information of the first peak. The adaptive spectrum of the EWT-WT method is shown in Figure 8d, which shows that the cutoff frequency between the noise and ECG signals is 0.667 Hz. The spectral segmentation boundary of EWT-WT adaptively varies with the different input signals. 

The real ECG signals collected by the non-contact ECG monitoring system were analyzed. Figure 9 shows the results of using the DWT, EMD, EWT and EWT-WT methods to reduce motion artifact for real ECG signals. Compared with the original ECG signal, the ECG signal denoised by the EWT-WT and EWT method retains the QRS waveform of the ECG signal, while the signal denoised by the DWT and EMD method has greater distortion. This shows that the EWT-WT and EWT method can remove motion artifact and improve the signal quality of real ECG signals. Compared with the EWT based method, the proposed EWT-WT based method can retain the R-peaks of the ECG signals, as shown in Figure 9d,e.

In Figure 10, it is observed that the amplitude of the Q wave of the original ECG signal is significantly lower than the amplitude of the S wave. However, the amplitude of the Q wave of the ECG signal denoised by the DWT and EMD method is slightly higher than that of S wave. This indicates ECG signals denoised by the DWT and EMD method show a significant distortion. The QRS wave is well restored in the ECG signal denoised by the EWT-WT and EWT motion artifact removal method. We can observe from Figure 10 that the denoised ECG signals by the EWT-WT and EWT method basically coincide with the original ECG signal, while the S wave and T wave of the denoised ECG signals using the DWT and EMD methods show a significant downward shift. The DWT and EMD based approaches fail to estimate the morphologies of QRS complexes of the ECG signals. The EWT-WT and EWT methods show better behavior in removing motion artifacts. The EWT-WT method has better ability to preserve the useful information for the ECG signals. The proposed EWT-WT method offers better performances than other methods recently published [8,10,29].

## 5. Conclusions

In this paper, we present a method based on empirical wavelet transform and wavelet thresholding for motion artifact removal from ECG signals. The aim of this work was to find an effective way to eliminate the motion artifacts in ECG signals for non-contact ECG monitoring systems. The proposed EWT-WT motion artifact removal method was applied on the simulated noise ECG signal, evaluated by qualitative and quantitative analysis, and compared with the DWT, EMD and EWT removal motion artifact methods. The results show that the EWT-WT motion artifact removal method has superior performance in restorage of the QRS complexes of original ECG signals, reducing distortion and retaining the useful information in ECG signals. As a filter, the proposed methodology can significantly improve the *SNR* value of the signal and effectively remove the motion artifact noise. Moreover, the experimental results of the EWT-WT motion artifact removal method for real ECG signals have shown its better ability to preserve the useful information for the ECG signals for non-contact ECG monitoring system. This methodology will be beneficial for the practical motion artifact elimination application in non-contact ECG monitoring systems.

## Figures and Tables

**Table 1:** The *MSE* of the output ECG signals using DWT, EMD, EWT and EWT-WT methods.

Record	DWT	EMD	EWT	EWT-WT
102	0.0050	0.0103	0.0017	0.0014
103	0.0077	0.0100	0.0019	0.0011
104	0.0041	0.0071	0.0013	0.0010
109	0.0085	0.0060	0.0036	0.0028
123	0.0018	0.0037	0.0011	0.0011
201	0.0135	0.0133	0.0025	0.0019
208	0.0146	0.0166	0.0045	0.0035
209	0.0029	0.0039	0.0014	0.0009
213	0.0041	0.0080	0.0030	0.0016
219	0.0124	0.0168	0.0024	0.0026
average	0.0075	0.0096	0.0023	0.0018

**Table 2:** The *PRD* of the denoised ECG signals using DWT, EMD, EWT and EWT-WT methods.

Record	DWT	EMD	EWT	EWT-WT
102	68.06	97.89	39.80	35.65
103	70.59	80.53	34.85	27.11
104	66.68	87.57	37.78	33.25
109	54.24	45.72	35.10	31.16
123	48.09	69.47	37.25	38.58
201	85.67	85.06	37.23	32.52
208	73.65	78.49	40.81	35.91
209	55.82	65.02	39.01	30.89
213	41.56	58.12	35.76	26.23
219	80.69	93.98	35.91	36.87
average	64.51	76.19	37.35	32.82

**Table 3:** The *SNR_imp_* comparison of the denoising results of using DWT, EMD, EWT and EWT-WT methods.

Record	DWT	EMD	EWT	EWT-WT
102	3.34	0.18	8.00	8.96
103	3.02	1.88	9.15	11.34
104	3.52	1.15	8.45	9.56
109	5.31	6.80	9.09	10.13
123	6.36	3.16	8.58	8.27
201	1.34	1.40	8.58	9.76
208	2.66	2.10	7.78	8.89
209	5.06	3.74	8.18	10.2
213	7.63	4.71	8.93	11.62
219	1.86	0.54	8.90	8.67
average	4.01	2.57	8.56	9.74
